# Temperature-sensitive albino gene *TCD5*, encoding a monooxygenase, affects chloroplast development at low temperatures

**DOI:** 10.1093/jxb/erw287

**Published:** 2016-08-16

**Authors:** Yufeng Wang, Jianhui Zhang, Xiaoliang Shi, Yu Peng, Ping Li, Dongzhi Lin, Yanjun Dong, Sheng Teng

**Affiliations:** ^1^Institute of Plant Physiology and Ecology, Shanghai Institute for Biological Sciences, Chinese Academy of Sciences, Shanghai 200032, China; ^2^Development Centre of Plant Germplasm Resources, College of Life and Environmental Sciences, Shanghai Normal University, Shanghai 200234, China

**Keywords:** Albino, chloroplast development, map-based cloning, monooxygenase, rice, temperature-sensitive.

## Abstract

Chloroplasts are essential for photosynthesis and play critical roles in plant development. In this study, we characterized the temperature-sensitive chlorophyll-deficient rice mutant *tcd5*, which develops albino leaves at low temperatures (20 °C) and normal green leaves at high temperatures (32 °C). The development of chloroplasts and etioplasts is impaired in *tcd5* plants at 20 °C, and the temperature-sensitive period for the albino phenotype is the P4 stage of leaf development. The development of thylakoid membranes is arrested at the mid-P4 stage in *tcd5* plants at 20 °C. We performed positional cloning of *TCD5* and then complementation and knock-down experiments, and the results showed that the transcript *LOC_Os05g34040.1* from the *LOC_Os05g34040* gene corresponded to the *tcd5* phenotype. *TCD5* encodes a conserved plastid-targeted monooxygenase family protein which has not been previously reported associated with a temperature-sensitive albino phenotype in plants. *TCD5* is abundantly expressed in young leaves and immature spikes, and low temperatures increased this expression. The transcription of some genes involved in plastid transcription/translation and photosynthesis varied in the *tcd5* mutant. Although the phenotype and temperature dependence of the *TCD5* orthologous mutant phenotype were different in rice and Arabidopsis, *OsTCD5* could rescue the phenotype of the Arabidopsis mutant, suggesting that *TCD5* function is conserved between monocots and dicots.

## Highlight

A new temperature-sensitive albino gene, *TCD5*, encoding a monooxygenase, affects chloroplast development at P4 stage under low temperature in rice.

## Introduction

Chloroplasts are defining features that distinguish plant cells from animal cells. They are the exclusive site of photosynthesis in higher plants, and are also responsible for the biosynthesis and storage of various metabolites (Jensen and Leister, 2014). The formation of a photosynthetically active chloroplast from a proplastid is controlled by both the plastid and nuclear genomes and accompanied by the rapid development of the thylakoid membrane; this is influenced by ambient conditions such as light and temperature (Mullet, 1993; López-Juez, 2007). Increasing evidence has suggested that chloroplast biogenesis is a highly regulated process.

Low temperatures are a major abiotic source of stress during rice growth. However, the molecular mechanisms involved in low-temperature stress on chloroplast development are not fully understood. Temperature-sensitive leaf variegation and chlorophyll-deficient mutants have been used as powerful models to elucidate the genetic network of chloroplast development because the degree of leaf variegation is regulated by developmental and environmental cues. In addition, leaf variegation and chlorophyll-deficient mutants are powerful tools that can be used to gain insight into the mechanisms underlying chloroplast biogenesis and chlorophyll (Chl) biosynthesis and metabolism (Sakamoto, 2003; Yu *et al.*, 2007). In rice, numerous leaf variegation mutants have been discovered and these are classified according to their phenotype as albino, chlorina, stripe (st), virescent (v) and zebra (http://www.shigen.nig.ac.jp/rice/oryzabase/top/top.jsp). Among these mutants, only a minority are temperature sensitive, including *virescent-1* (*v1*), *virescent-2* (*v2*), *virescent-3* (*v3*), *stripe-1* (*st1*), *v*5, *v*7, *chs*1, *chs*2, *chs*3, *chs*4, *chs*5, *Fan5*, *7436S*, *al12*, *Cde1 (t*), *tsc (t*), *mr21*, *ysa*, and *tws* (Iba *et al.*, 1991; Dong *et al.*, 2001; Xia *et al.*, 2006; Liu *et al.*, 2007; Su *et al.*, 2012). The phenotype of these mutants is normal or near normal at permissive temperatures but abnormal under higher or lower temperatures. The rice mutants *v1*, *v2*, and *v3* are early virescent mutants that develop albino leaves at restrictive low temperatures (20 °C) but nearly normal green leaves at permissive higher temperatures (32 °C), which suggests a temporal responsive factor that governs aberrant chloroplast development under low temperature conditions (Kusumi and Iba, 2014). The *V1* gene that encodes the chloroplast-localized protein NUS1 has been reported to play a role in the regulation of chloroplast rRNA metabolism (Kusumi *et al.*, 1997). NUS1 is involved in the establishment of the plastid genetic system during early chloroplast development under cold stress conditions (Kusumi *et al.*, 2011). The *V2* gene encodes a guanylate kinase (pt/mt GK) that regulates guanine nucleotide pools in developing leaves (Sugimoto *et al.*, 2007). The *V3* and *St1* genes encode the large and small subunits of ribonucleotide reductase (RNR), respectively, and reduced RNR activity can impair chloroplast DNA replication in developing leaves and ultimately lead to the virescent phenotype (Yoo *et al.*, 2009).

At present, a number of chlorophyll-deficient mutants are being studied in rice. *TCD9* encodes the α subunit of a housekeeping chaperonin Cpn60 protein involved in mediating the folding of newly synthesized, translocated, or stress-denatured proteins, and it is responsible for the albino phenotype of rice at low temperatures (Jiang *et al.*, 2014). Disruption of the rice genes *OsV4* and *YSA* encoding pentatricopeptide repeat proteins caused a seedling-specific albino phenotype at low temperatures (Su *et al.*, 2012; Gong *et al.*, 2014). *WLP1* encodes a 50S chloroplast ribosome L13 protein and is necessary for chloroplast development in rice; however, the leaf and immature panicle of its mutant are albino at low temperatures (Song *et al.*, 2014).

Despite the discovery of a number of genes involved in chloroplast development at low temperatures, the complex biogenesis of this organelle is not well understood. In this study, we report the identification and characterization of the rice mutant *tcd5* (temperature-sensitive chlorophyll-deficient mutant 5) from a ^60^Co-irradiated population. Physiological and molecular analyses suggest that the *TCD5* gene is essential for chloroplast differentiation during early leaf development under cold stress.

## Materials and methods

### Plant material and growth conditions

The mutant *tcd5* was isolated from a ^60^Co-irradiated mutant pool of the japonica rice cultivar Jiahua1. The *tcd5* and wild-type (WT) Jiahua1 plants were grown in a paddy field in Shanghai, China (31°11′N) during the summer season or in a growth chamber. The phenotype of young leaf chlorosis has been stably inherited for more than three generations in the field in Shanghai. Plants were grown in a paddy field in Shanghai to evaluate the primary agronomic traits during the rice growing season of 2013. Means from three replications were calculated and Student’s *t* test was performed for statistical analysis.

For temperature treatments, seedlings of *tcd5* and WT plants were cultivated on filter paper with sterile water in growth chambers with 600 μmol m^−2^ s^−1^ light intensity, 14h light–10h dark photoperiod, 70% moisture and different constant temperatures (20, 24, 28 and 32 °C).

*Arabidospis thaliana* ecotype Columbia (Col) and its confirmed homozygous T-DNA insertion line SALK_059716 were obtained from Arabidopsis Biological Resource Center (ABRC). Seeds of Arabidopsis were sterilized by 5% sodium hypochlorite solution for 10min and sown on MS medium with 0.8% agar. Arabidopsis were grown in the 22 °C chamber under a 16h light–8h dark photoperiod at 100 μmol m^−2^ s^−1^, with 50% relative humidity. For temperature treatments, sterilized seeds were sown on MS medium and cultured at different constant temperatures (19, 22 and 28 °C). Photographs were taken 8 d later when plants were in the seedling stage.

### Measurement of photosynthetic pigment

Both the chlorophyll and carotenoid contents were measured using a spectrophotometer according to the method of Arnon (1949) with minor modifications. Equal weights (0.5g) of freshly collected second top leaves were immersed in 95% ethanol for 48h under dark conditions. Residual plant debris was removed by centrifugation. The supernatants were analysed with a DU 800 UV/Vis Spectrophotometer (Beckman Coulter, USA) at 663, 645 and 470nm. The mean values of three biological replicates were calculated.

### Protein extraction, BN/SDS-PAGE and western blotting

Equal weights (1.0g) of fresh leaves were collected and ground to a fine powder in liquid nitrogen, then homogenized with 5ml solubilizing buffer containing 0.1M Tris–HCl, pH 6.8, 2% SDS, 0.6% (v/v) 2-mercaptoethanol, 10% (v/v) glycerol, and protease inhibitor cocktail (Sigma-Aldrich, USA) (1:100). The homogenate was incubated at 70 °C for 5min and centrifuged at 10 000×*g* for 5min. The supernatant was the total protein, which was quantified by a BCA protein quantification kit (Shanghai Yeasen Biotechnology Co., Ltd, China). Equal amounts (20 μg) of total protein were loaded onto 10% SDS–polyacrylamide gels and separated by electrophoresis. Then the proteins were transferred to a PVDF membrane (Millipore, USA) and incubated with MYC-tagged specific antibodies. The signals were detected using an ECL Plus Western Blotting Detection Kit (GE Healthcare, USA) and visualized using a Tanon 5500 imaging system (Shanghai Tannon Co., Ltd, China).

The isolation of thylakoid membranes was performed as described (Huang *et al.*, 2013). The detergent dodecylmaltoside was added to thylakoid membrane samples at 2% (w/v) concentration. After solubilizing for 30min on ice, the samples were centrifuged for 20min at 16 000×*g* at 4 °C. Each lane was loaded with an equal amount of Chl (5 μg). After performing blue native (BN) PAGE, the gels were photographed or stained with Coomassie staining solution (Wittig *et al.*, 2006).

### Transmission electron microscopy

Leaf samples were fixed in 2.5% (v/v) glutaraldehyde (0.2M phosphate buffer, pH 7.2) at 4 °C for 16h. After separately rinsing with phosphate buffer three times, the samples were treated with 1% (w/v) osmium tetroxide at 4 °C for 4h and washed with phosphate buffer again. Then, the samples were dehydrated through an ethanol series [50%, 70%, 85%, 95%, and 100% (v/v)]. Ethanol was subsequently replaced by a series of Spurr’s resin dilutions [25%, 50%, 75%, and 100% (v/v)]. Finally, the samples were embedded in Spurr’s resin at 65 °C for 16h. Ultrathin sections of the samples were cut with a diamond knife, and collected on copper grids. The sections were then stained with uranyl acetate and observed using a Hitachi H-7650 transmission electron microscope (Hitachi Ltd, Japan).

### Temperature-shift experiments

The temperature-shift experiments were performed under high (32 °C) and low (20 °C) temperatures with 600 μmol m^−2^ s^−1^ light intensity, 14h light–10h dark, 70% moisture. For shift-down experiment, the WT and *tcd5* seeds were sown in water under high temperature, and one group (ten seedlings each of WT and *tcd5*) was transferred each day to the low temperature for continuous culture. The date that the experiments were initiated was labelled day 0. The relative Chl contents in fully expanded third leaves of every seedling in each group of treatment were measured using a SPAD-502 meter (Minolta Co., Japan). The SPAD value was read five times per leaf. The average SPAD value and standard deviation were calculated. The shift-up experiments (from low temperature to high temperature) were conducted similarly.

### Map-based cloning of the *TCD5* gene

For the genetic analysis, a cross was performed between the *tcd5* mutant and PA64S cultivar (indica variety). The F_2_ population of 1309 plants with the *tcd5* mutant phenotype was used for fine mapping. Sequence polymorphisms between Nipponbare and 9311 (Indica) were identified and used to develop molecular markers, such as SSR and InDel. Primer pairs were designed using Primer 5.0. The newly developed PCR-based molecular markers used in this study are listed in Supplementary Table S1 at *JXB* online. The PCR procedure was as follows: 95 °C for 5min; 35 cycles of 95 °C for 30s; 50–55 °C annealing for 30s; 72 °C for 40s; and a final elongation step at 72 °C for 5min.

### Vector construction and plant transformation

For the complementation vector, a 2163-bp cDNA fragment containing the entire *TCD5* (*LOC_Os05g34040.1*) open reading frame (ORF) was amplified by the overlap extension PCR method with TCD5 p1s/p2a and TCD5 p3s/p4a primers (see Supplementary Table S2) using Prime STAR HS DNA Polymerase (TaKaRa, Japan) from cDNA of Jiahua1. For the *LOC_Os05g34040.2* transcripts, we ordered the clone AK065380 from Rice Genome Resource Center (RGRC; http://www.rgrc.dna.affrc.go.jp/). After verification by sequencing, fragments of *TCD5* (*LOC_Os05g34040.1*) and AK065380 (*LOC_Os05g34040.2*) were separately amplified using attB-TCD5-cds and attB-AK380-cds primers and inserted into the pDONR207 vector using the Gateway BP recombination reaction, and then recombined into pGWB17 (Karimi *et al.*, 2007) to produce CaMV35-driven TCD5-MYC or AK065380-MYC plant expression vectors by the LR reaction. For the RNAi recombinant vector, a 478bp fragment of *LOC_Os05g34040* was amplified with the primer pair RNAi-TCD5 (see Supplementary Table S2). The fragment was inserted into pTCK303 (Stoutjesdijk, 2002; Wu *et al.*, 2007) to form the construct RNAi-TCD5.

The recombinant constructs pGWB17-TCD5 and pGWB17-AK065380 were introduced into Agrobacterium EHA105 and then used to transform *tcd5* according to a published method (Hiei *et al.*, 1994). Agrobacterium EHA105 containing RNAi-TCD5 was used to transform Nipponbare. The pGWB17-TCD5 and pGWB17-AK065380 were introduced into Agrobacterium GV3101 and transformed into SALK_059716 using the floral dip method (Clough and Bent, 1998). All transformants were confirmed by hygromycin resistance (50mg l^–1^) and PCR tests using the HYB primer (see Supplementary Table S2).

### Sequence alignment and phylogenetic analysis

Gene predictions and structure analyses were performed using databases from the National Centre for Biotechnology Information (NCBI). Monooxygenase sequences in rice were identified using the GRAMENE database (www.gramene.org/). A BLAST analysis was performed on the NCBI website to search for homologues of TCD5. Multiple sequence alignments were conducted and a phylogenetic tree was constructed using MEGA v4.1 software. Bootstrap replication (1000 times) was used for statistical support for the nodes in the phylogenetic tree.

### RNA isolation, reverse transcription, RT-PCR and qPCR

Total RNA was extracted using the TRIzol method following the manufacturer’s instructions (Aidlab Biotechnology Co., Ltd, China). The first-strand cDNA was synthesized using a First Strand cDNA Synthesis Kit (Toyobo, Japan). RT-PCR was carried out using MR40-RT1 and actin primers (Supplementary Table S2). qRT-PCR was conducted using the Bio-Rad IQ5 Real Time system with the SYBR Green Mix following the manufacturer’s instructions (TaKaRa, Japan) with specific primers (Supplementary Table S3). For each sample, qRT-PCR was performed with three technical replications on three biological replicates. The 2^−∆∆*C*T^ method was used to analyse these genes’ relative changes (Livak and Schmittgen, 2001). Student’s *t* test was performed for statistical analysis.

### Subcellular localization

The initial 399-bp coding sequence of *TCD5* was amplified using the specific primer PA-TCD5 (see Supplementary Table S2) and cloned into the N-terminus of green fluorescent protein (GFP) under the control of the CaMV35S promoter in the transient expression vector pA7-GFP (created by Dr Katrin Czempinski, Potsdam University, Germany). The pA7-GFP and recombinant vector pA7-TCD5-GFP were then transformed into Arabidopsis protoplasts according to previously described protocols (Yoo *et al.*, 2007). GFP fluorescence was visualized using an Olympus FV1000 confocal laser-scanning microscope.

## Results

### Identification of the temperature-sensitive chlorophyll-deficient mutant *tcd5*

**Fig. 1.**
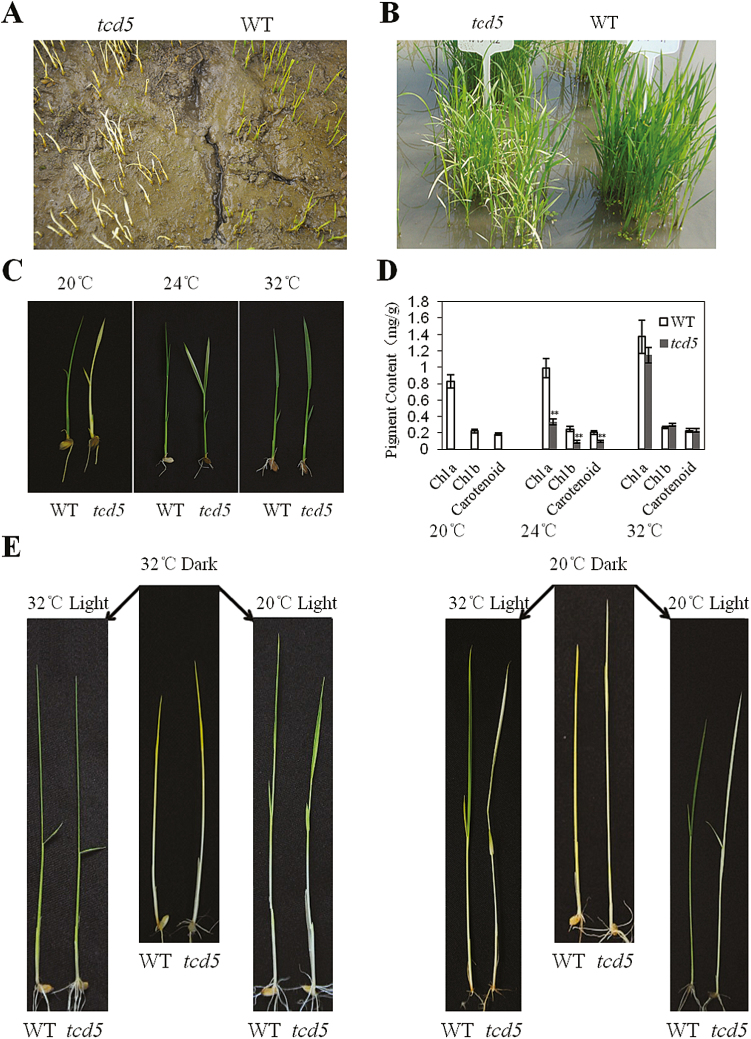
Phenotypic characteristics of the wild-type (WT) Jiahua1 and *tcd5* mutant. (A) Phenotype of the WT and *tcd5* plants germinated in the field. (B) Phenotype of the WT and *tcd5* plants at the seedling stage in the field. (C) Phenotype of the WT and *tcd5* seedlings in different temperature chambers. (D) Leaf pigment contents of the WT and *tcd5* seedlings in different temperature chambers. **Highly significant at *P*<0.01 by Student’s *t* test. (E) Phenotype of the WT and *tcd5* seedlings transferred from dark conditions to light conditions. The seedlings were cultured in dark conditions in different temperature chambers and then transferred to light conditions at a different temperature. Photographs were taken 2 days after transfer.

The rice (*Oryza sativa* L.) temperature-sensitive chlorophyll-deficient mutant *tcd5* was isolated from a ^60^Co-irradiated population of *japonica* variety Jiahua1. Under field conditions, the *tcd5* homozygous mutant showed the albino phenotype after germination (Fig. 1A), and the new leaves had green–white variegated stripes at the bottom and an albino tip at the seedling stage (Fig. 1B). At the tillering stage, the newly developed leaves were nearly the same as the wild-type Jiahua1 (WT). The phenotype of *tcd5* did not show obvious differences compared with the WT at the mature stage (see Supplementary Fig. S1). The WT and *tcd5* plants had similar agricultural traits, and only the effective tiller number per plant and grain weight per plant in the *tcd5* plants were reduced compared with the wild-type (Table 1).

**Table 1.** *Primary agronomic traits of the* tcd5 *mutant and WT Jiahua1 grown in fields*

Plant variety	Plant height (cm)	No. of effective tillers per plant	Spike length (cm)	Grain weight per plant (g)	Setting rate	1000-grain weight (g)
WT	101.25±4.72	10.42±1.50*	17.39±0.57	33.01±4.78*	0.93±0.010	24.51±0.61
*tcd5*	98.88±5.13	8.0±0.63*	16.92±1.53	27.62±3.97*	0.93±0.022	23.94±0.74

Values are the mean±SD, *n*=12. *Significant at *P*<0.05 by Student’s *t* test.

To verify whether the phenotypic variation of the *tcd5* mutant depended on the developmental stage or the low temperature conditions in the early period of the growth season (20–22 °C at the seedling stage), we planted *tcd5* and WT seeds in growth chambers under different constant temperatures. Under 20 °C conditions, the *tcd5* mutant produced fully albino leaves and sheaths that were never restored (Fig. 1C). In addition, chlorophyll (Chl) was undetectable in the leaves (Fig. 1D), and the *tcd5* mutant gradually died when the fourth or the fifth leaf blade fully emerged from the sheath. Under the 24 °C condition, the *tcd5* mutant developed green–white leaves and sheaths; the upper and middle parts of the leaves were white, whereas the basal half was green (Fig. 1C). The Chl *a* and Chl *b* contents were 34% and 37% of the contents in the WT, respectively, and the carotenoid content was approximately 47% of the WT (Fig. 1D). The *tcd5* mutant exhibited nearly the same phenotype as the WT at 32 °C (Fig. 1C) and presented similar Chl and carotenoid contents (Fig. 1D). These results indicated that the albino phenotype of *tcd5* was dependent on the temperature.

In the dark at 20 and 32 °C, etiolated seedlings were obtained from the WT under both temperature conditions, whereas the *tcd5* seedlings were etiolated at 32 °C but albino at 20 °C (Fig. 1E). These seedlings were then transferred to light conditions at 20 °C and 32 °C, respectively. After 2 days, the etiolated WT and *tcd5* seedlings turned green at either 20 °C or 32 °C, but the albino *tcd5* seedlings raised at 20 °C remained albino at 20 °C and 32 °C (Fig. 1E).

### Chloroplast and etioplast development was affected at low temperatures in the *tcd5* mutant

**Fig. 2.**
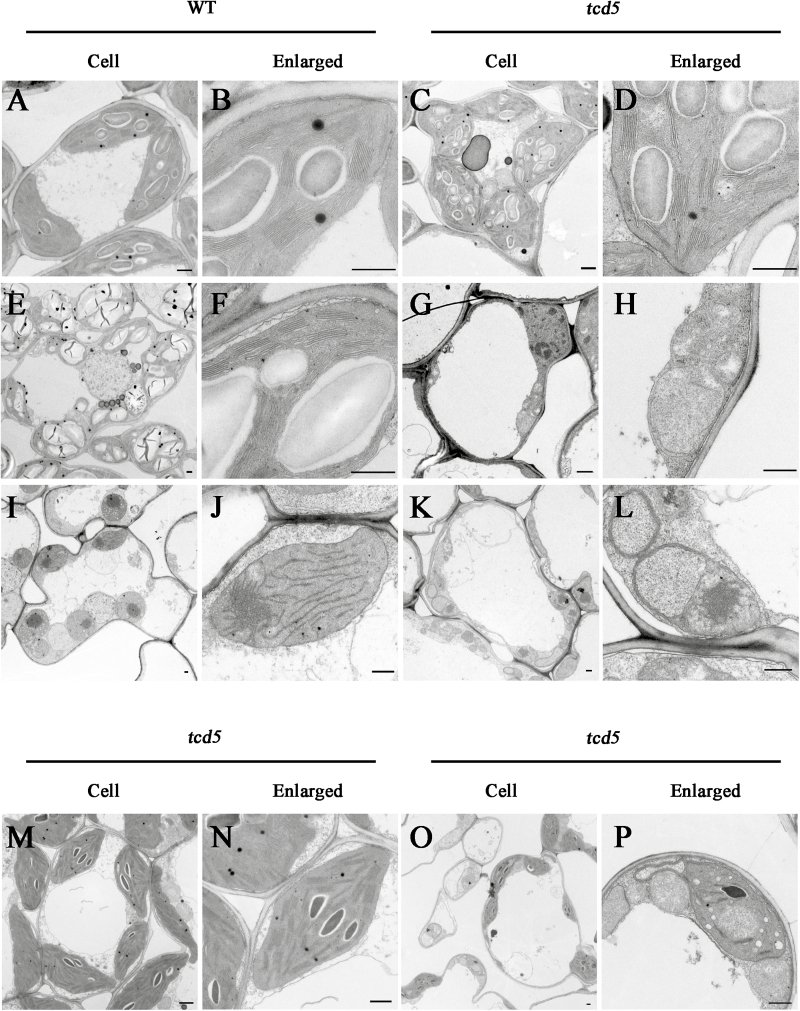
Transmission electron microscopy images of cells from WT and *tcd5* mutants grown under different temperature conditions. Cells and chloroplast structures from WT (A, B) and *tcd5* (C, D) at 32 °C; WT (E, F) and *tcd5* (G, H) at 20 °C; WT (I, G) and *tcd5* (K, L) at 20 °C in the dark; the green part of the *tcd5* green–white leaf (M, N); and the albino part of the green–white leaf (O, P) at 24 °C. All the cells were from the third leaf of the seedlings at the three-leaf stage. Scale bar: 0.5 μm.

The ultrastructure of the chloroplasts in the mesophyll cells of the WT and *tcd5* plants was examined by transmission electron microscopy (TEM). At 32 °C, the WT plant displayed normal chloroplasts with well-developed lamellar structures equipped with normally stacked grana and thylakoid membranes (Fig. 2A, B), and the *tcd5* plants also displayed chloroplasts with well-developed lamellar structures equipped similarly to that of the WT (Fig. 2C, D). At 20 °C, however, the WT developed a number of large starch grains and chloroplasts with normal thylakoids (Fig. 2E, F), whereas the albino leaf cells in *tcd5* contained few starch grains and fewer chloroplasts. The chloroplasts appeared less well developed and lacked well-structured thylakoid membranes (Fig. 2G, H). Under 32 °C dark conditions, the chloroplasts in the *tcd5* plant occurred in the form of an etioplast, which had a similar size and complete lattice structure to the chloroplasts in the WT plant (data not shown). Under 20 °C dark conditions, the WT still developed a normal etioplast with a complete or assembling lattice structure (Fig. 2I, J), whereas the *tcd5* showed a relatively reduced number of etioplasts, with certain etioplasts lacking internal membrane structures and some etioplasts only forming a primary lattice structure (Fig. 2K, L).

To investigate the development of the chloroplast in the green–white leaves of *tcd5*, we examined the ultrastructure of *tcd5* mutants at 24 °C. Cells from the green part of the blade appeared normal, and the chloroplasts had a regularly stacked grana and thylakoid structure (Fig. 2M, N). Mesophyll cells from the albino region had dramatically fewer chloroplasts, although certain chloroplasts contained fewer granal thylakoids or few lamellar internal membrane structures (Fig. 2O, P).

### Formation of photosynthetic complexes is impaired at low temperatures in the *tcd5* mutant

**Fig. 3.**
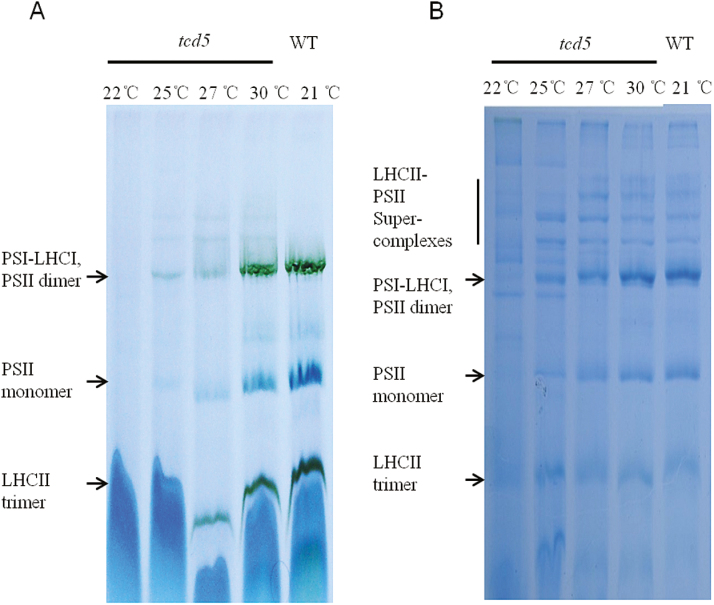
Blue native PAGE of the WT and *tcd5* plants under different temperatures. Total thylakoid protein complexes were isolated from leaves of the WT and *tcd5* mutant grown in different temperature chambers. Proteins were separated by blue native PAGE and photographed (A) or stained with Coomassie Brilliant Blue (B). Each lane was loaded with 5μg Chl.

We further investigated the effect of temperature on the photosynthetic complexes in the thylakoid membranes. Blue native PAGE analyses did not detect the photosynthetic complexes in the mutant at 22 °C (Fig. 3A, B). When the temperature was above 25 °C, PSII super-complexes, PSI–LHCI, PSII dimers, PSII monomers and LHCII trimers could be detected, and the amounts were lower than that in the WT grown at 21 °C (Fig. 3A, B). With increases in temperature, the amounts of the photosynthetic proteins increased, and the amounts were similar to the WT when the growth temperature reached 30 °C. This result indicated that low temperature affects the formation of photosynthetic complexes in the *tcd5* mutant.

### P4 stage is the temperature-sensitive period for the *tcd5* mutant

In rice, the leaf primordial and leaf emergence stages are synchronized. The rice leaf development processes are defined as a series of successive stages as follows: P0 (leaf founder), P1 (leaf primordium), P2 (hood-shaped primordium), P3 (ligule primordium), P4 (rapid elongation of leaf blade), P5 (rapid elongation of leaf sheath), and P6 (a fully expanded leaf) (Itoh *et al.*, 2005; Kusumi *et al.*, 2010*a*). With the synchronized production of the leaf primordial stage (plastochron) and leaf emergence stage (phyllochron), the developmental stage of the plant can be deduced according to the stage of the emerged leaf (Itoh *et al.*, 2005; Kusumi *et al.*, 2011). Therefore, the critical leaf developmental stage at which low temperatures produce the albino phenotype can be determined. Hence, temperature-shift experiments were performed to determine the temperature-sensitive period (TSP) of leaf development (Iba *et al.*, 1991).

**Fig. 4.**
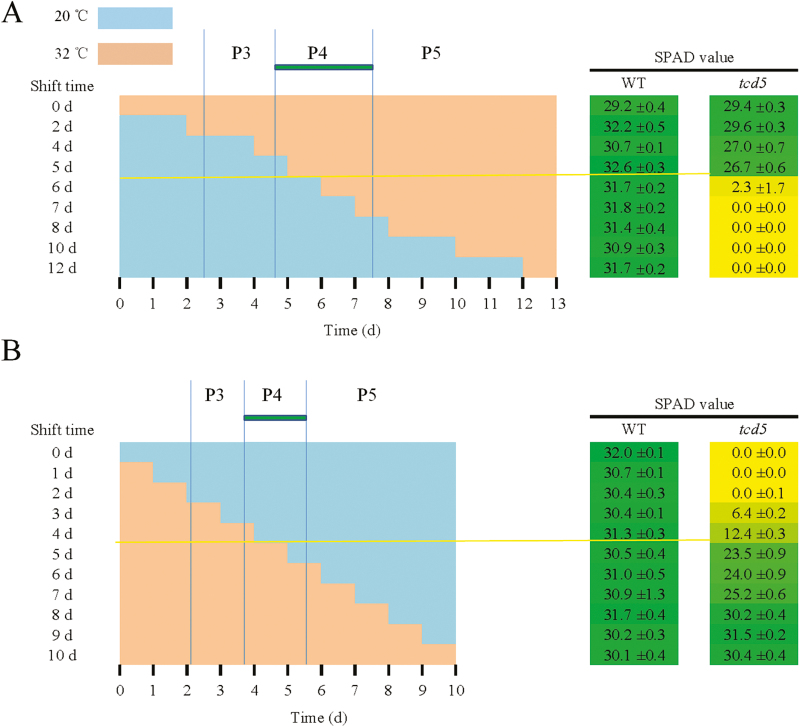
Temperature-shift experiments. Shift-up experiment (A) and shift-down experiment (B). Pale red and pale blue represent the high temperature (32 °C) and the low temperature (20 °C), respectively. The SPAD value of each group is shown on the right and corresponds to the growth treatment.

Leaf SPAD value represents the relative Chl content (Monje and Bugbee, 1992). The SPAD value of the third leaves in the WT was high and hardly affected by the temperature shift. In the shift-up experiments, the average time of emergence of the first, second and third blades of *tcd5* from the sheath was 2.6, 4.7, and 7.6 d, respectively, at which time the third blade had just entered the P3, P4, and P5 stages, respectively, at 20 °C. High SPAD values were detected in the third leaves of the *tcd5* seedlings that were grown at low temperatures for 0–5 d, and the SPAD values were dramatically decreased to extremely low levels when the shift occurred after the emergence of the second leaf when the third leaf was entering the P4 stage; however, a temperature shift before or after this period did not affect the relative chloroplast content (Fig. 4). In the shift-down experiments, the first, second and third blades of *tcd5* emerged at 2.1, 3.7, and 5.6 d, respectively, at which time the third blade had just entered the P3, P4, and P5 stages, respectively, at 32 °C. Low SPAD values were detected in the third leaves of the *tcd5* seedlings that were grown at 32 °C for 0–4 d, and SPAD values were dramatically increased to high levels when the shift occurred after the emergence of the second leaf when the third leaf was entering the P4 stage. However, shifts before or after this period did not affect the relative chloroplast content (Fig. 4). These results indicated that the TSP in the *tca5* mutant is the P4 stage.

**Fig. 5.**
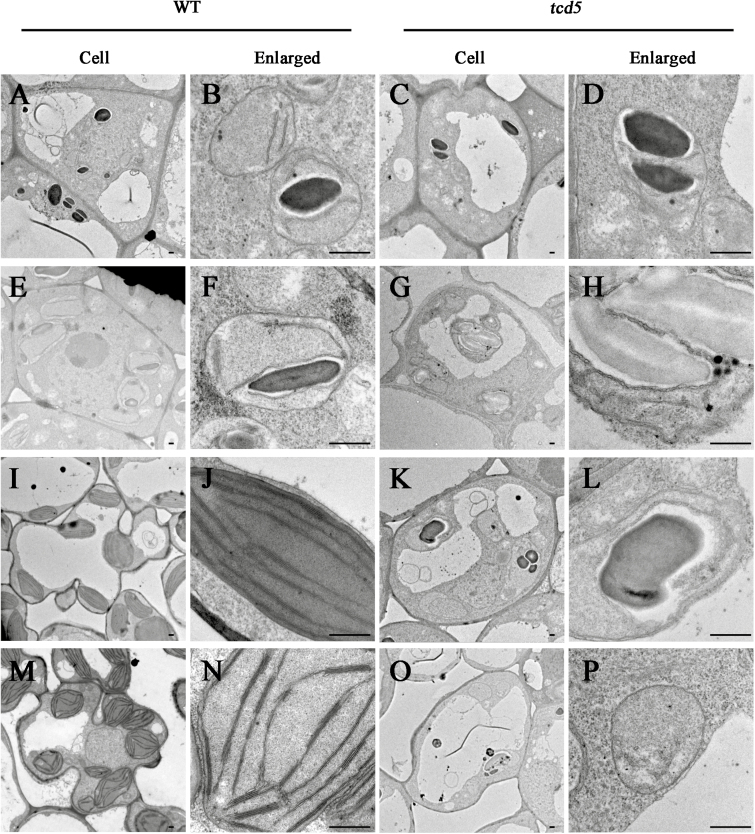
Transmission electron microscopy images of the cells from the WT and *tcd5* chloroplasts from the developing leaves at the P4 stage. Cells and chloroplast structures from the WT (A, B) and *tcd5* plants (C, D) at the P4-2 stage, WT (E, F) and *tcd5* plants (G, H) at the P4-4 stage, WT (I, G) and *tcd5* plants (K, L) at the P4-5 stage, and WT (M, N) and *tcd5* plants (O, P) at the P4-6 stage. All the cells were from the fourth leaf of seedlings at the three-leaf stage. Scale bar: 0.5 μm.

The P4 stage was divided into several sub-stages designated as P4-*X*, where *X* indicates the leaf length (Kusumi *et al.*, 2010*a*). P4 leaves had an initial length of 1–3mm and reached a final size of about 6cm in three-leaf stage WT and *tcd5* plants at 20 °C. Detailed ultrastructure at different P4 sub-stages was further investigated by TEM. At the P4-2 stage, slight differences in the plastid structures between the WT (Fig. 5A, B) and *tcd5* plants (Fig. 5C, D) were observed. In the WT, the plastids contained starch grains and several poor internal membrane structures, whereas in the *tcd5* plants, the plastids mainly contained starch grains and only a few internal membrane structures in certain cells. The differences between the WT and *tcd5* plants were more severe in the development process. At the P4-4 stage, starch grains were small or absent and the thylakoid membrane system was developed in the WT chloroplasts (Fig. 5E, F), whereas the plastids still contained large starch grains and had only developed several internal membrane structures in the *tcd5* plants (Fig. 5G, H). During the P4-5 stage, thylakoid extension and grana formation in the chloroplasts were observed in the WT (Fig. 5I, J), whereas the internal membranes were not developed and could not be differentiated into grana and stroma thylakoids in the *tcd5* plants (Fig. 5K, L). At the P4-6 stage, thylakoid extension and grana thylakoid formation were observed in the chloroplasts of the WT plants (Fig. 5M, N), whereas the plastids contained small starch grains and the internal membranes were not observed in the *tcd5* mutant (Fig. 5O, P). These results verified that the *tcd5* mutation affected thylakoid membrane differentiation, which led to disorders of chloroplast biogenesis at the P4 stage at low temperatures.

### Genetic analysis and map-based cloning of *TCD5*

The genetic basis of the temperature-dependent albino phenotype was analysed. All F_1_ hybrids from the cross between the *tcd5* mutant (as the maternal material) and the WT exhibited normal green leaves at 20 °C. Among the 845 F_2_ individuals, 598 green individuals and 247 albino individuals were identified at 20 °C. Therefore, a three to one (χ^2^=0.97, χ^2^ 0.05=3.84) segregation ratio was evident. The reverse hybridization between the WT (as the maternal material) and *tcd5* mutant also showed a three to one separation ratio in the 1128 F_2_ individuals (881 green individuals and 247 albino individuals, χ^2^=1.68). These results indicated that the temperature-dependent albino phenotype was controlled by a single pair of recessive genes.

**Fig. 6.**
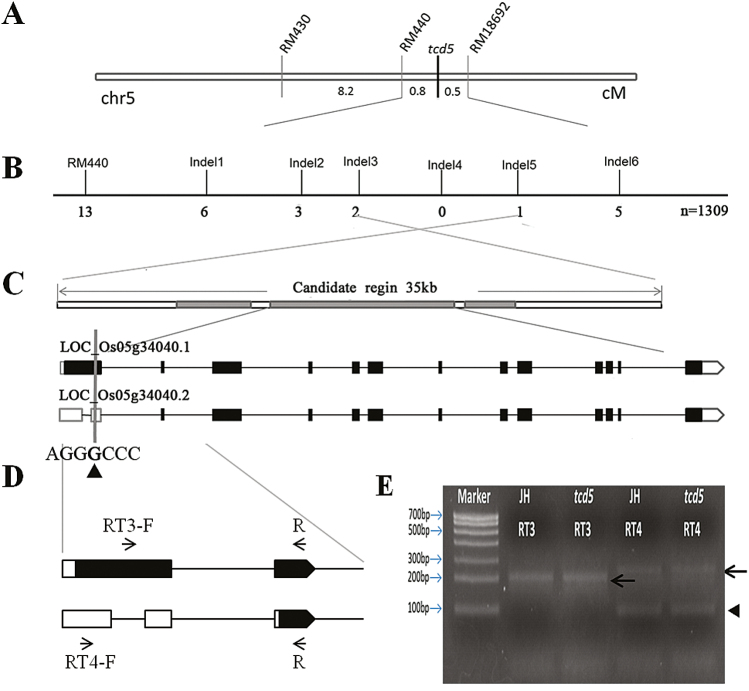
Genetic mapping of the *TCD5* locus. (A) Preliminary map of the *TCD5* locus between RM440 and RM18692. Numbers below indicate the genetic distance (cM) between the markers. (B) Fine mapping of the *TCD5* locus. (C) One candidate gene (*LOC_Os05g34040*) was found to contain a 1bp deletion. The triangle shows the position of the deleted G in *TCD5*. Intervening lines indicate introns, and boxes indicate exons. Black boxes indicate ORFs, whereas white boxes indicate the 5′-UTR and 3′-UTR. (D) Schematic illustration of the specific primers RT3 and RT4. (E) PCR amplification of RT3 and RT4 from WT (JH) and *tcd5*. Two expected fragments (200bp, 228bp) from *LOC_Os05g34040.1* were amplified using RT3 and RT4 from JH and *tcd5*. The arrow indicates the two fragments. The triangle indicates the non-specific band of the expected size (162bp) from *LOC_Os05g34040.2*.

Genetic mapping was performed using the F_2_ population generated from the *tcd5* mutant and PA64S cultivar. Using SSR markers and 40 F_2_ individuals, the *TCD5* locus was initially mapped between RM440 and RM18602 on chromosome 5 (Fig. 6A). Then, a large F_2_ population with 1309 individuals derived from the same cross was used for fine mapping (Fig. 6B). Six InDel markers were developed between RM440 and RM18602 (see Supplementary Table S1), and the *TCD5* locus was finally narrowed to a 35kb interval between InDel 3 and InDel 5 in the BAC clone P0668F02 (GenBank accession number: AC130729.3). Three expressed genes in this region were previously identified by the Rice Genome Annotation Project (rice.plantbiology.msu.edu). Only a single base (G) deletion in *LOC_Os05g34040* was found in the *tcd5* mutant (Fig. 6C).

The genome sequence of *LOC_Os05g34040* contains 9498 nucleotides and there are two gene models: *LOC_Os05g34040.1* (containing 13 exons and 12 introns), and *LOC_Os05g34040.2* (containing 14 exons and 13 introns). The ORF of *LOC_Os05g34040.1* starting from the first exon encodes a polypeptide of 721 amino acids. The G deletion at 392bp of *LOC_Os05g34040.1* CDS terminates translation in advance and results in a 130 aa peptide in the *tcd5* mutant (Fig. 6C). The ORF of *LOC_Os05g34040.2* starts from the third exon and encodes a polypeptide of 583 amino acids, although it lacks the 138 amino acids at the N terminus compared with the *LOC_Os05g34040.1* protein. *LOC_Os05g34040.2* is supported by the full-length cDNA clone AK065380. We designed the specific primers RT3 and RT4 (see Supplementary Table S2) to verify the presence of these two transcripts. Two expected fragments from *LOC_Os05g34040.1* were amplified from the WT and *tcd5* mutant and verified by a sequence analysis (Fig. 6D). Although one fragment of the expected size was obtained from *LOC_Os05g34040.2*, the sequence analysis indicated that it was an unspecific product (Fig. 6E). This result indicates that only *LOC_Os05g34040.1* was detected.

### *TCD5* encodes *LOC_Os05g34040.1*

**Fig. 7.**
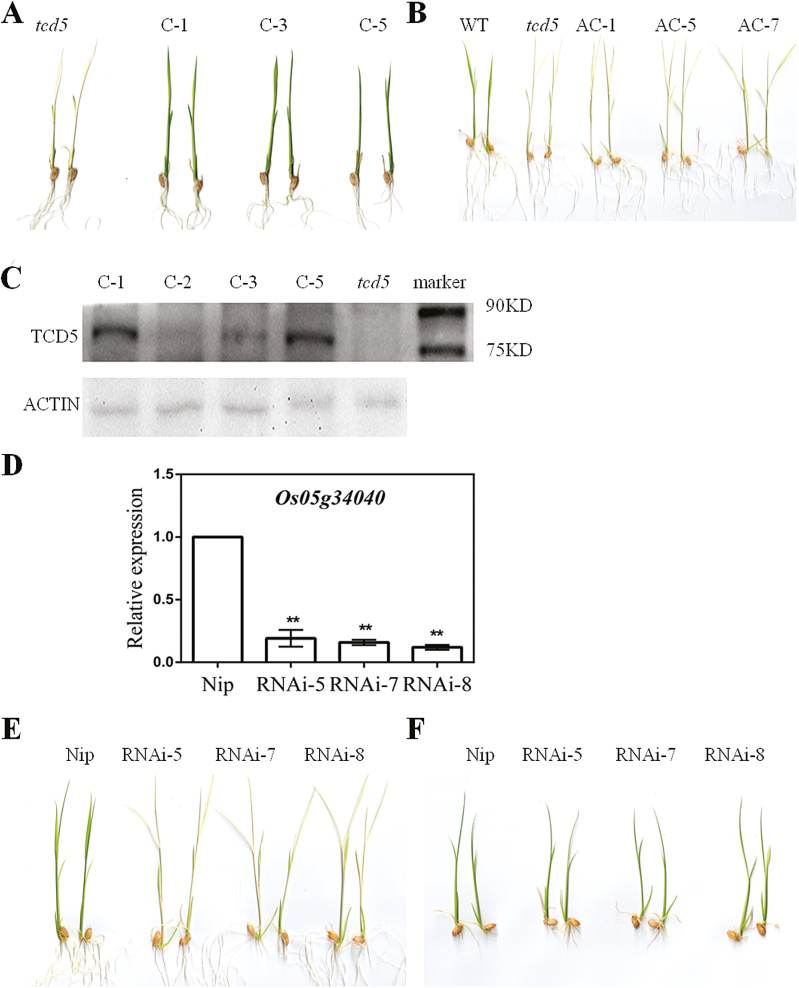
Functional complementation analysis of the *TCD5* allele. (A) Phenotype of the *tcd5* and *LOC_Os05g34040.1* complemented plants (C-1, C-3 and C-5) at 20 °C. (B) Phenotype of the WT and *LOC_Os05g34040.2* complemented plants (AC-1, AC-5 and AC-7) at 20 °C. (C) Western blot analysis of the imported TCD5-MYC protein in the *tcd5* and *LOC_Os05g34040.1* complemented lines (C-1, C-2, C-3 and C-5) at 20 °C using a MYC antibody. The fusion protein TCD5-MYC was approximately 79kDa. Actin was used as the internal reference. (D) Relative expression of *TCD5* in the Nipponbare (Nip) and LOC_Os05g34040-RNAi lines at 20 °C by qPCR (mean±SD, *n*=3). The expression level in Nip was set to 1. **Highly significant compared with Nip at *P*<0.01 by *t* test. (E) Phenotype of the Nip and LOC_Os05g34040-RNAi lines (RNAi-5, RNAi-7 and RNAi-8) at 20 °C. (F) Phenotype of the Nip and LOC_Os05g34040-RNAi lines (RNAi-5, RNAi-7 and RNAi-8) at 32 °C.

The T-DNA binary vectors with CaMV35S promoter-driven *LOC_Os05g34040.1* or *LOC_Os05g34040.2* were constructed and transformed into *tcd5* mutants. At 20 °C, the independent transgenic lines with *LOC_Os05g34040.1* (C-1, C-3, and C-5) presented a restoration of the green phenotype from the albino phenotype (Fig. 7A), whereas the transgenic plants with *LOC_Os05g3404.2* (AC-1, AC-5, and AC-7) retained the albino phenotype (Fig. 7B). The protein expression of exogenous MYC-fusion of TCD5 in the transgenic lines was verified by western blotting (Fig. 7C). The transcript amounts of *LOC_Os05g34040* were higher in *LOC_Os05g34040.2* transgenic lines by qPCR, which indicated the expression of exogenous *LOC_Os05g34040.2* (see Supplementary Fig. S2). The *LOC_Os05g34040* knock-down transgenic lines in the Nipponbare background were also obtained. The amount of *LOC_Os05g34040* was obviously decreased in the RNAi lines (RNAi-5, RNAi-7 and RNAi-8) by qPCR (Fig. 7D), which developed white or pale-green leaves at 20 °C and normal green leaves at 32 °C (Fig. 7E, F). Thus, we concluded that *LOC_Os05g34040* is responsible for *TCD5* and *LOC_Os05g34040.1* is the active transcript.

### *TCD5* is strongly expressed in young tissue and up-regulated by low temperature

**Fig. 8.**
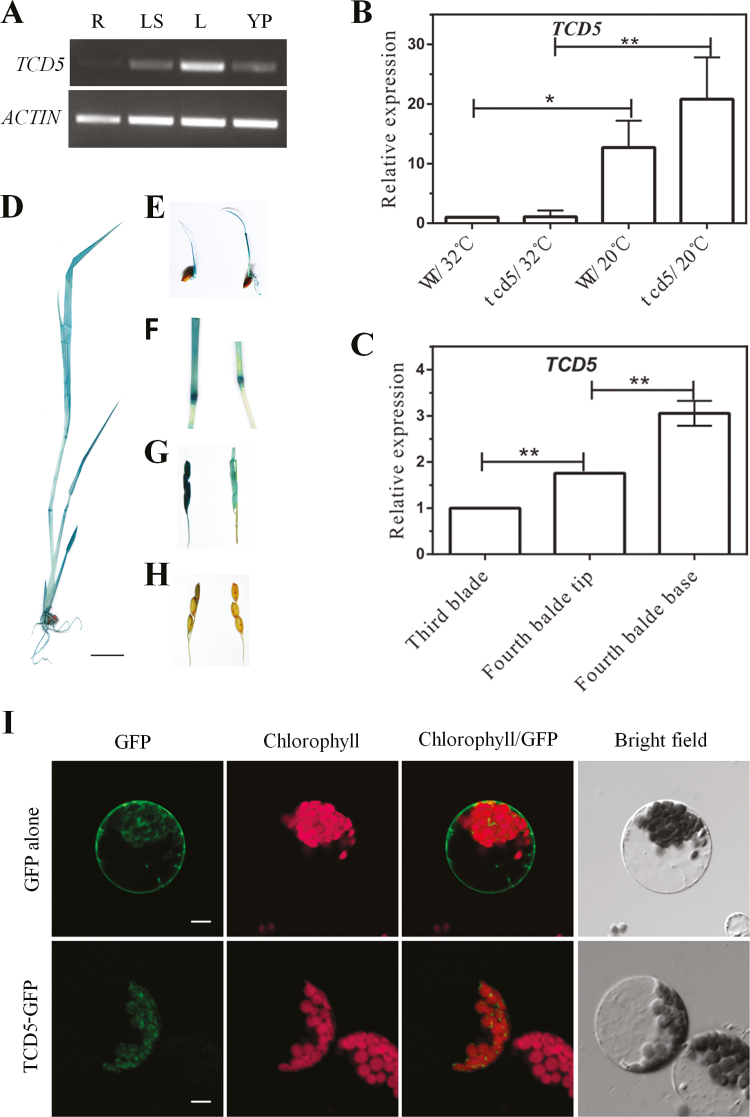
Expression pattern of *TCD5*. RT-PCR analysis of the *TCD5* transcripts in the organs of rice. Total RNA was isolated from the following tissues: roots (R), leaves (L), leaf sheath (LS) and young panicle (YP). (B) qPCR analysis of *TCD5* in the leaves of the WT and *tcd5* plants at 20 or 32 °C at the three-leaf stage (mean±SD, *n*=3). The expression level in WT at 32 °C was set to 1. **Highly significant compared with WT at *P*<0.01 by *t* test. (C) qPCR analysis of the *TCD5* gene in different leaves and different parts of the leaf in the WT plant at 20 °C at the four-leaf stage (mean±SD, *n*=3). The expression level in the third leaf was set to 1. **Highly significant compared with the third blade at *P*<0.01 by one-way ANOVA plus Dunnett’s *t* test. GUS staining of four-leaf seedlings (D), germinated seedlings (E), young internodes (F), young spikes (G) and mature stems and seeds (H) in the TCD5 promoter:GUS transgenic plants. Scale bar: 1.0cm. (I) Chloroplast localization of TCD5. GFP alone and TCD5:GFP were transiently expressed in Arabidopsis protoplasts. Scale bar: 5 μm.

We examined the expression levels of *TCD5* in different organs of WT rice by RT-PCR. *TCD5* was strongly expressed in the leaves, and it was also expressed in the leaf sheath and young panicle and weakly expressed in the root (Fig. 8A). We further investigated the relative expression of *TCD5* in leaves at 20 °C and 32 °C by qPCR. The transcript abundance of *TCD5* at 20 °C was higher than that at 32 °C both in WT and the *tcd5* mutant (Fig. 8B). In different leaf blades and different parts of the leaf blades, we found that younger blades contained a greater amount of the *TCD5* transcript than the older blade and the tip of the blade contained a greater amount of the *TCD5* transcript than the base (Fig. 8C).

We also developed transgenic plants with *TCD5* promoter-driven β-glucuronidase (GUS) to detect the expression pattern of *TCD5* in detail. GUS activity was first observed when the transformed lines germinated. The roots and leaves from 4-d-old seedlings to four-leaf stage seedlings were all stained (Fig. 8D, E). As the plant grew, the GUS staining degrees decreased in the roots, sheaths, and leaves, and almost no stain was observed in the stem and the mature leaf blade. Blue dye was observed in the young internode and young spike (Fig. 8F, G), and high levels of GUS activity were observed in the youngest spike, although the levels decreased following the development of the spike and were undetectable in the mature seed (Fig. 8H).

### TCD5 protein located in the chloroplast

Twenty-five amino acid residues at the N-terminus of the TCD5 protein were predicted to constitute a chloroplast transit peptide by SignalP software (http://www.cbs.dtu.dk/services/SignalP/). To determine the precise intracellular localization of TCD5, a construct with a CaMV35S-driven TCD5–GFP fusion protein was generated using the pA7 vector and transiently expressed in Arabidopsis mesophyll cells. The green fluorescence signals from GFP were present in the cytoplasm and the membrane of cells transformed with a control construct (CaMV35S:GFP) (Fig. 8I). The green fluorescence signals from CaMV35S:TCD5-GFP co-localized with chloroplasts that showed red auto-fluorescence signals. These data indicated that TCD5 was a plastid protein.

### *TDC5* affects the transcription of genes associated with chloroplast development at low temperatures

**Fig. 9.**
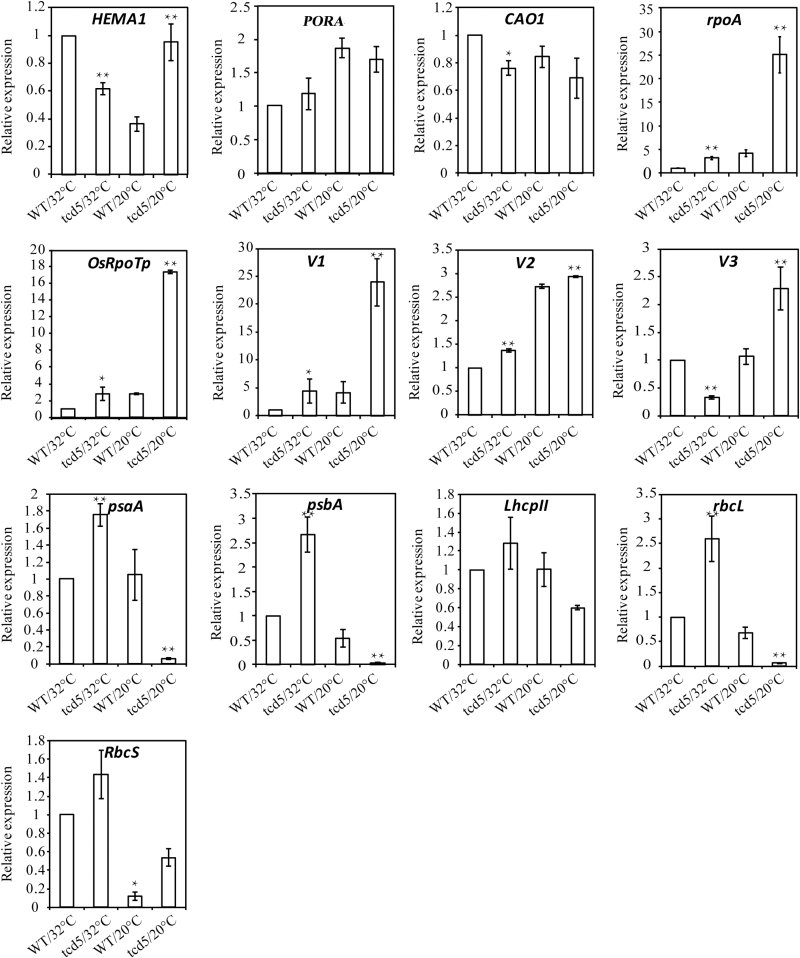
Transcription of genes associated with chlorophyll synthesis, chloroplast development and photosynthesis. Expression analysis of the *HEMA1*, *PORA*, *CAO1*, *OsRpoTp*, *rpoA*, *V1*, *V2*, *V3*, *LhcpII*, *psaA*, *psbA*, *rbcL*, and *RbcS* genes by qPCR. RNA was isolated from the third leaf of seedlings at the three-leaf stage. Values are the mean±SD from three independent biological replicates. The expression level in WT at 32 °C was set to 1. *P<0.05, **P<0.01 by *t* test.

To investigate the change in expression of genes associated with Chl biosynthesis and chloroplast development in the mutant, we performed an additional qPCR analysis (Fig. 9).

The chlorophyll synthesis genes *HEMA1* (glutamyl tRNA reductase), *CAO1* (chlorophyllide a oxygenase), and *PORA* (encoding NADPH-dependent protochlorophyllide oxidoreductase) in the mutant had similar transcription levels to those of the wild-type at 20 °C and *HEMA1* was significantly up-regulated. These results indicated that Chl deficiency in *tcd5* was not caused by the down-regulated expression of these Chl synthetic genes.

*OsRpoTp* and *rpoA* encode the nuclear-encoded RNA polymerase (NEP) and p-encoded RNA polymerase (PEP) α subunit, respectively, which are involved in the regulation of chloroplast transcription/translation activity. *V1*, *V2*, and *V3* function in the establishment of the plastid genetic system. Compared with the wild-type, *OsRpoTp*, *rpoA*, and *V1* were significantly up-regulated at 20 and 32 °C in the mutant. The expression of *V2* was slightly upregulated in the *tcd5* mutant. *V3* was up-regulated under 20 °C but down-regulated under 32 °C.

However, the expression levels of the photosynthesis-related genes *LhcpII* (light harvesting Chl *a*/*b*-binding protein of PSII), *rbcL* (the large subunit of Rubisco) and *psaA* and *psbA* (two reaction centre polypeptides) were significantly reduced in the *tcd5* mutant at 20 °C with the exception of the nuclear *RbcS* gene (small subunit of Rubisco) (Fig. 9). This result indicates that the photosynthesis components were seriously impaired at low temperatures in the *tcd5* mutant.

Taken together, the loss of *TCD5* function led to a massive disordered expression of the genes associated with chloroplast biogenesis at low temperatures.

### Molecular function of *TCD5* is conserved between rice and Arabidopsis

TCD5 was annotated as a putative monooxygenase with an unknown function by the Rice Genome Annotation Project (http://rice.plantbiology.msu.edu/) (Kawahara *et al.*, 2013). The functional domain analysis revealed that TCD5 contains a pyridine nucleotide-disulfide oxidoreductase conserved domain (240–327 aa) and an uncharacterized FAD-dependent dehydrogenase multi-domain (140–699 aa). A paralogue of TCD5 does not occur in the rice genome.

Forty-two monooxygenases in rice can be divided into six subgroups. An evolutionary analysis of the monooxygenase protein sequences showed that TCD5 was located distal to the other oxygenases (see Supplementary Fig. S3).

**Fig. 10.**
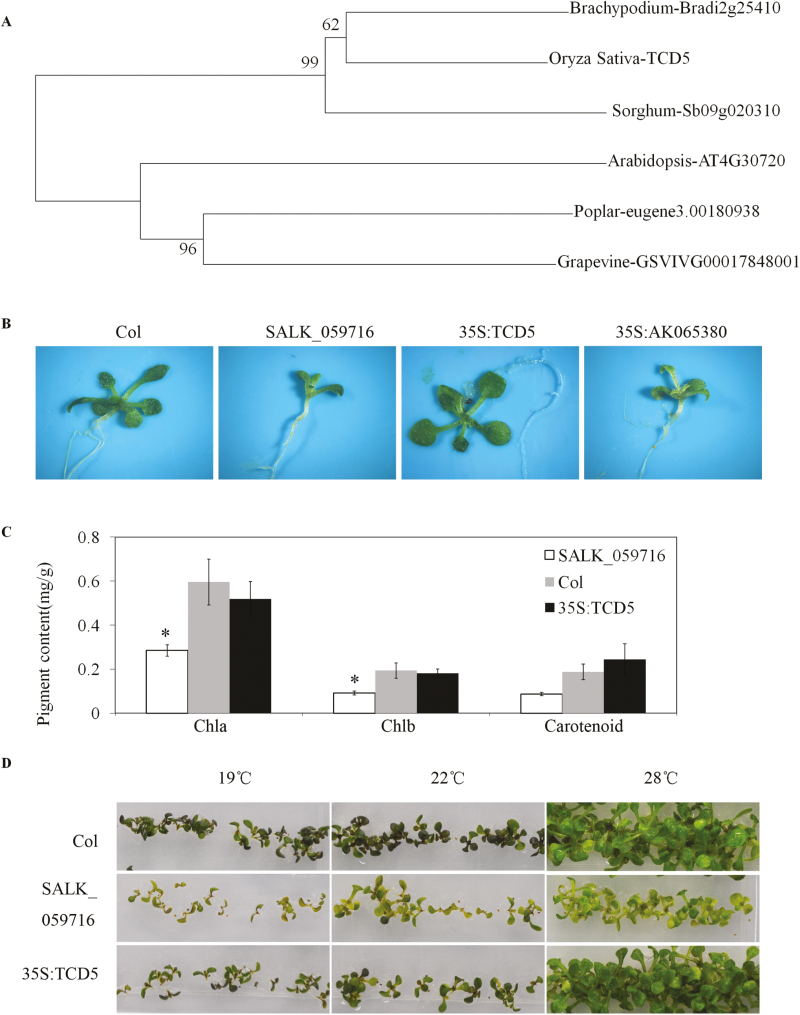
*OsTCD5* could complement the phenotype of the At4g30720 mutant in Arabidopsis. (A) Phylogenetic analysis of TCD5 and its orthologue proteins by the neighbour-joining method. Bootstrap values are shown at the nodes. (B) Phenotype of the Arabidopsis Col wild-type, At4g30720 mutant (SALK_059716), *OsTCD5* complemented SALK_059716 line (35S:TCD5) and *LOC_Os05g34040.2* complemented SALK_059716 line (35S: AK065380) plants in the 22 °C greenhouse. (C) Chl content analysis of the Col, SALK_059716 and 35S:TCD5 line plants in the 22 °C greenhouse. *Significant compared with Col at *P*<0.05 by one-way ANOVA plus Dunnett’s *t* test. (D) Phenotype of the Col, SALK_059716 and 35S:TCD5 line plants on MS at different temperatures (19, 22, and 28 °C).

Orthologues of TCD5 have been found in other plant species, such as *Brachypodium distachyon*, *Aegilops tauschii*, *Sorghum bicolor*, *Arabidopsis thaliana*, and *Camelina sativa* (Fig. 10A). The rice TCD5 protein shared 67% identity with its Arabidopsis orthologue, At4G30720. The functions of the *TCD5* orthologues were all uncharacterized except for At4G30720 (Vlad *et al.*, 2010), which belongs to the FAD/NAD-binding oxidoreductase family, and its protein is also located in the chloroplast (Vlad *et al.*, 2010). The Arabidopsis T-DNA insertion mutant SALK_059716, in which T-DNA is inserted in the fourth exon of At4g30720, showed a smaller size and pale green leaf (Vlad *et al.*, 2010). The 35S:TCD5 and 35S:AK065380 were constructions that were also transformed into SALK_059716. Transgenic lines with 35S:TCD5 restored the Col phenotype; however, the phenotype of transgenic lines with *35S:*AK065380 were not different from those of SALK_059716 (Fig. 10B). The Chl content of the 35S:TCD5 complement line was similar to that of Col, although the content of SALK_059716 was obviously lower than that of Col (Fig. 10C). The temperature dependence of the SALK_059716 phenotype was further investigated. Eight days after germination, the SALK_059716 plants were smaller than the Col plants, especially at 19 °C. SALK_059716 still showed paler green leaves than Col under any of the temperature conditions (Fig. 10D), and the SALK_059716 phenotype could not be recovered, even at 28 °C, which is a high temperature for Arabidopsis. These results indicate that in addition to differences in the phenotype between the *tcd5* and SALK_059716 plants, the temperature dependence of the phenotype is also different.

## Discussion

### Monooxygenase gene *TCD5* is essential for chloroplast development at low temperatures in rice

Rice is more susceptible to cold stress than other cereals, such as wheat and barley. In field conditions, sudden low-temperature periods often occur in spring during early seedling development. Therefore, it is reasonable to infer that rice has developed a mechanism to ensure that leaf and internal chloroplast development is not hindered by low temperature-induced retardation (Kusumi and Iba, 2014). The identification of mutants with a low temperature albino phenotype and cloning of the relevant genes are extremely important processes for elucidating the mechanisms underlying chloroplast development at low temperatures. To date, low temperature-sensitive albino rice genes have been cloned, including *V1*, *V2*, *V3*, *ST1*, *OsV4*, *WLP1*, and *TCD9*. These genes primarily function in DNA replication and repair; nucleic acid metabolism; RNA splicing, editing and maturation; and protein folding (Kusumi *et al.*, 1997; Sugimoto *et al.*, 2004; Yoo *et al.*, 2009; Jiang *et al.*, 2014; Song *et al.*, 2014). In this work, we isolated and characterized a new rice temperature-sensitive albino mutant, *tcd5*, which presents a phenotype similar to the reported *v1*, *v2*, and *v3* mutants. The *tcd5* mutant produced albino leaves at low temperatures (20 °C) but normal leaves at higher temperatures (32 °C). These changes in leaf colour were accompanied by changes in the chlorophyll content and chloroplast development. Positional cloning of *tcd5* showed that transcript *LOC_Os05g34040.1* corresponded to the phenotype, and this result was verified by complementation and knock-down experiments. *TCD5* putatively encodes a monooxygenase family protein targeted to the plastid. The involvement of this type of protein has not been previously reported for the low temperature albino phenotype in rice or other plant species.

Similar to other low temperature-sensitive albino genes, *TCD5* was expressed in most tissues, and the expression was especially high in young tissues. In the *tcd5* mutant, the genes involved in Chl synthesis were not down-regulated, implying that *TCD5* does not affect the expression of genes in the pathway of chlorophyll synthesis at low temperatures. However, the genes involved in chloroplast development were dramatically altered. Significant increases were observed in NEP and PEP, which participate in transcribing the plastid gene expression machinery and activating photosynthetic apparatus expression, which is consistent with the function of the V1 and V3 genes. Genes functioning in the establishment of the photosynthetic apparatus, including *psaA*, *psbA*, *LhcpII* and *RbcL*, were obviously inhibited. It is interesting that the expression pattern of these genes in *tcd5* was similar to that of *v1*, *v2*, *v3* and *st1* (Kusumi *et al.*, 1997; Sugimoto *et al.*, 2004; Yoo *et al.*, 2009), which suggested the presence of a common mechanism that mediates chloroplast protein expression and assembly at low temperatures in these mutants.

### *TCD5* functions at the second step of chloroplast differentiation at low temperatures

The process of chloroplast development from the proplastid to functional chloroplasts is synchronous with the process of leaf development in rice (Kusumi *et al.*, 2010*b*). Chloroplast differentiation can be divided into three steps (Mullet, 1993): the first step involves the activation of plastid replication and plastid DNA synthesis and begins at the P0–P3 stages and finishes at the early P4 stage (Kusumi and Iba, 2014) (see Supplementary Fig. S4); the second step involves the establishment of the chloroplast genetic system and occurs at the P4 stage (Kusumi *et al.*, 2010*a*), with NEP preferentially transcribing the plastid gene expression machinery (Hajdukiewicz *et al.*, 1997) (Supplementary Fig. S4); the final step involves the high expression of plastid and nuclear genes, which encode the photosynthetic apparatus, thereby leading to the synthesis and assembly of the photosynthetic machinery (Yagi and Shiina, 2014), and it occurs from the late P4 to P5 stage (Kusumi and Iba, 2014) (Supplementary Fig. S4). Our results showed that the TSP of the *tcd5* mutant was the P4 stage, which was similar to that of the *v1*, *v2*, and *v3* mutants. The TEM analysis indicated that chloroplast development was impaired in the early to mid-P4 stage at low temperatures. The apparent abnormality of chloroplast differentiation was first demonstrated at the mid-P4 phase. Analyses of the accumulation of chloroplast transcripts in the *tcd5* mutant revealed that the chloroplast genetic system was not reduced at the transcription level at low temperatures and the following step of chloroplast development was disturbed because of the defective transcription of the photosynthetic machinery-related genes. The enhancement to the chloroplast genetic system related to gene expression might have been caused by feedback regulation related to the defective transcription of photosynthetic machinery-related genes. Therefore, the TSP of *tcd5* is the P4 stage, and *TCD5* functions in the second step of chloroplast differentiation at low temperatures.

### TCD5 orthologues have conserved molecular functions but show diverse mutants’ phenotypes

*TCD5* encodes an atypical monooxygenase that is different from other monooxygenase genes in rice, which implies that *TCD5* may not have evolved from a common ancestor and may have other functions compared with known oxygenase family members. Interestingly, this gene is conserved in higher plants. Orthologues of TCD5 can be found in both monocots and dicots. The orthologue protein of TCD5 in Arabidopsis putatively functions in photosynthetic chain electron flow, and the mutant presented a pale green and growth-defective phenotype, although the development of the chloroplast was unaffected (Vlad *et al.*, 2010). The leaf colour phenotype in the Arabidopsis mutant was temperature independent. These results indicate that the phenotypes and the thermo dependence of the phenotypes of the mutants of these two orthologues were different in rice and *Arabidopsis*. Surprisingly, the rice *TCD5* gene could rescue the pale green and growth-defective phenotype of the Arabidopsis mutant, indicating relative conservation in the molecular function of *TCD5* between dicots and monocots. Studies of this new class of low-temperature albino genes will help demonstrate the mechanism of chloroplast development under low temperatures.

## Supplementary data

Supplementary data are available at *JXB* online.

Table S1. Primers used for fine mapping.

Table S2. Primers used for plasmid construction and RT-PCR.

Table S3. Primers used for real-time PCR.

Figure S1. Phenotypes of WT and *tcd5* at the mature stage in the field.

Figure S2. Relative expression of the *TCD5* gene in the *LOC_Os05g34040.2* complemented plants (AC-1, AC-5 and AC-7) at 20 °C by qPCR.

Figure S3. Phylogenetic analysis of the TCD5 protein in rice by the neighbour-joining method.

Figure S4. Schematic illustration of a rice seedling and chronological progression of the principal growth steps during leaf development (Kusumi *et al.*, 2010*a*).

Supplementary Data
