# Supplementary material for: Temperature-sensitive albino gene TCD5, encoding a monooxygenase, affects chloroplast development at low temperatures
Source: J Exp Bot. 2016 Aug 16;67(17):5187–202. doi: 10.1093/jxb/erw287 (PMC5014166; doi:10.1093/jxb/erw287)
Supplement: Supplementary Data [file supp_67_17_5187__index.html]

Temperature-sensitive albino gene TCD5, encoding a monooxygenase, affects chloroplast development at low temperatures — Temperature-sensitive albino gene TCD5, encoding a monooxygenase, affects chloroplast development at low temperatures — Supplementary Data 

# Temperature-sensitive albino gene *TCD5*, encoding a monooxygenase, affects chloroplast development at low temperatures

## Supplementary Data

Data files

- supplementary\_tables\_S1\_S3\_figures\_S1\_S4.pdf - Supplementary Data
