# Supplementary material for: Temperature-sensitive albino gene TCD5, encoding a monooxygenase, affects chloroplast development at low temperatures
Source: J Exp Bot. 2016 Aug 16;67(17):5187–202. doi: 10.1093/jxb/erw287 (PMC5014166; doi:10.1093/jxb/erw287)
Supplement: Supplementary Data [file supp_erw287_supplementary_tables_S1_S3_figures_S1_S4.pdf]

Temperature-sensitive albino gene *TCD5*, encoding a monooxygenase, affects chloroplast development at low temperatures

Yufeng Wang[1], Jianhui Zhang[2], Xiaoliang Shi[1], Yu Peng[1], Ping Li[1], Dongzhi Lin[2], Yanjun Dong[2] and Sheng Teng[1]*

[1]Institute of Plant Physiology & Ecology, Shanghai Institute for Biological Sciences, Chinese Academy of Sciences, Shanghai 200032, China.
[2]Development Centre of Plant Germplasm Resources, College of Life and Environmental Sciences, Shanghai Normal University, Shanghai 200234, China.

Table S1 Primers used for fine mapping.

Primer pair name	Forward primer (5’-3’)	Reverse primer (5’-3’)
Indel 1	ACCTCTTTCGGTGCCGGTTTTTCCA	TCTACCATTCCCCTCTCGCTTCTCC
Indel 2	GTTGTTTTATAGAACCTGCACCTGT	ATCGAGATGTACTTTCAATAGCCTG
Indel 3	TCTTCCATCCTCCGCCATTGTTCTT	GCATCTCCATTTCTCACACGCTACT
Indel 4	AATAGTTCGGTTGGAGGGTTTGTGA	GCCGTTATTAGTTAGGAGTGTT
Indel 5	GATTGTATACGAATTCTCCACG	TTTGTAGTACTTGGGGTCCTATTCA
Indel 6	ACCGATGTTAGGTGGAGCAATGGCA	GATTGTCGCTGTGACCACCATTTCC

Table S2 Primers used for plasmid construction and RT-PCR.

Primer pair name	Forward primer (5'-3')	Reverse primer (5'-3')
RT 3	GATAGGCGACACAAGGAGCTGCT	TCAGGGAGCATAGAAGAAACCGG
RT4	GCGGTACCCGTCGGAGAAGAAG	TCAGGGAGCATAGAAGAAACCGG
TCD5-p1s/p2a	CGGGATCCGCTAAAACCCACATTATCCTCA	GGCGGGTTACTGGCGGCTCTCCA
TCD5-p3s/p4a	CGGGAATTTCAGTGCTTTGGCGA	AGGGTCGACACAAGATTAAAATGCCGTCCTC
attB-TCD5-cds	GGGGACAAGTTTGTACAAAAAAGCAGGCTTCATGGCGCTCGCCGCCGCCTC	GGGGACCACTTTGTACAAGAAAGCTGGGTCATATTTTACGAAGCCTTTTTG
attB-AK380-cds	GGGGACAAGTTTGTACAAAAAAGCAGGCTTCTCAAACCCTAGCCGTCCCAGCCTCG	GGGGACCACTTTGTACAAGAAAGCTGGGTCATATTTTACGAAGCCTTTTTG
RNAi-TCD5	GGGGTACCACTAGTGCTGTTAAGAGACTTCTGGATT	CGGGATCCGAGCTCACTACTAAGGCACCAATATCAC
PA7-TCD5	CCGCTCGAGATGGCGCTCGCCGCCGCCTCCGT	ACGCGTCGACGAATTTCAGTGCTTTGGCGATGG
HYB	TGAAAAAGCCTGAACTCACCG	TATTTCTTTGCCCTCGGACG
MR40-RT1	CCAGCCTCGGCTTGACCAAT	TCCGGAGCAGCTCCTTGTGT
actin	TGCTATGTACGTCGCCATCCAG	AATGAGTAACCACGCTCCGTCA

Table S3 Primers used for real-time PCR.

Primer pair name	Forward primer (5’-3’)	Reverse primer (5’-3’)
q-TCD5	CTCCCAACCAGACACTATCA	CGCCCTTTATGTACTTCAGC
OsRpoTp	TCCTCATGTCGAGCAAGGAT	GAAAGAATGTCTGGACTTTG
V2	TGTCTATGGGGAGTATAAGGGGA	AATCTCTCTCAAGGTTGCTGCTC
V3	ATCAGAGCCAGAGTCTTAATGTT	TGTTCGTAGATAATACATCCCAG
rbcS	TACTACGATGGCAGGTATTGGAC	GAAGCCGATGATGCGAACAAATG
rbcL	CTTGGCAGCATTCCGAGTAA	ACAACGGGCTCGATGTGATA
LhcpII	GAAGAAGATCAAGAACGGCC	TTGCCGGGGACGAAGTTGGT
psbA	AGAGACGCGAAAGTACAAGC	AAGTTGCGGTCAATAAGGTA
psaA	GCGAGCAAATAAAACACCTTTC	GTACCAGCTTAACGTGGGGAG
rpoA	GCGTCTTTATTATGGTCGTTTCA	CTCCCAAATTTTGCATGTGTGAT
HEMA1	TATCGTGGAACCGTGGGATTAGA	AGATACCTCAAACAGATGGCGTG
PORA	TGTACTGGAGCTGGAACAACAA	GAGCACAGCAAAATCCTAGACG
CAO1	GACTATCCCTTCTCTGCTGCCTC	GTGGATGTATGAGTAAAAGGAGC
NUS1	TCAGAACGAGAAGGATTCAGCA	GGCAACAGCCACTAAAATTTCT
actin	TGCTATGTACGTCGCCATCCAG	AATGAGTAACCACGCTCCGTCA

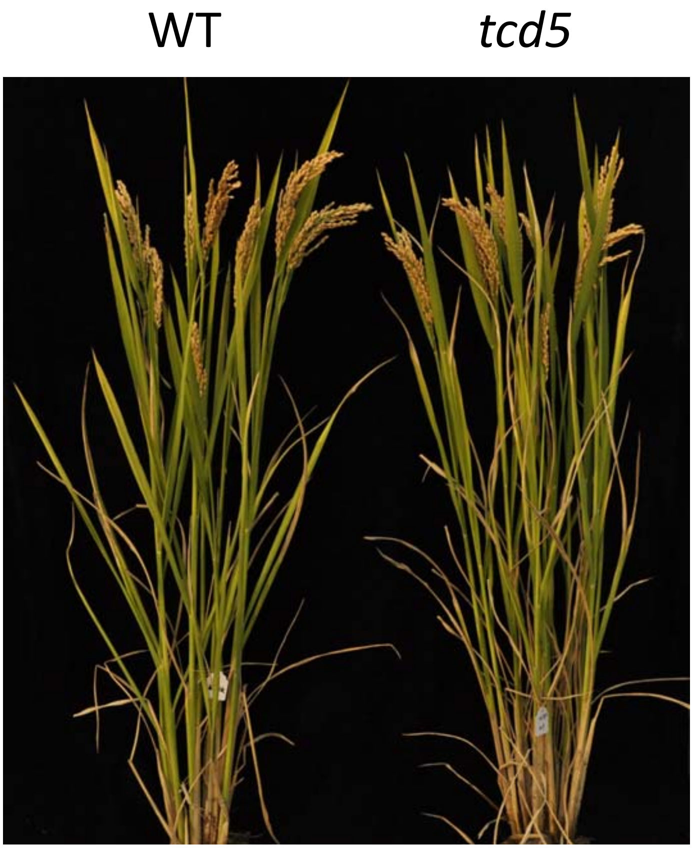

Fig. S1. Phenotypes of WT and *tcd5* at the mature stage in the field.

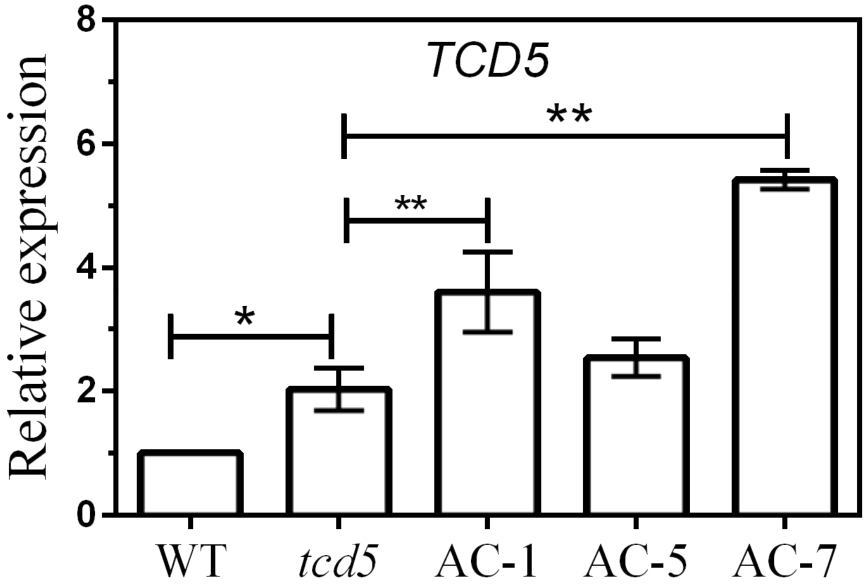

Fig. S2. Relative expression of the *TCD5* gene in the LOC_Os05g34040.2 complemented plants (AC-1, AC-5 and AC-7) at 20° C by qPCR.The expression level in *tcd5* mutant was set to 1. **, highly significance at P < 0.01 by T test.

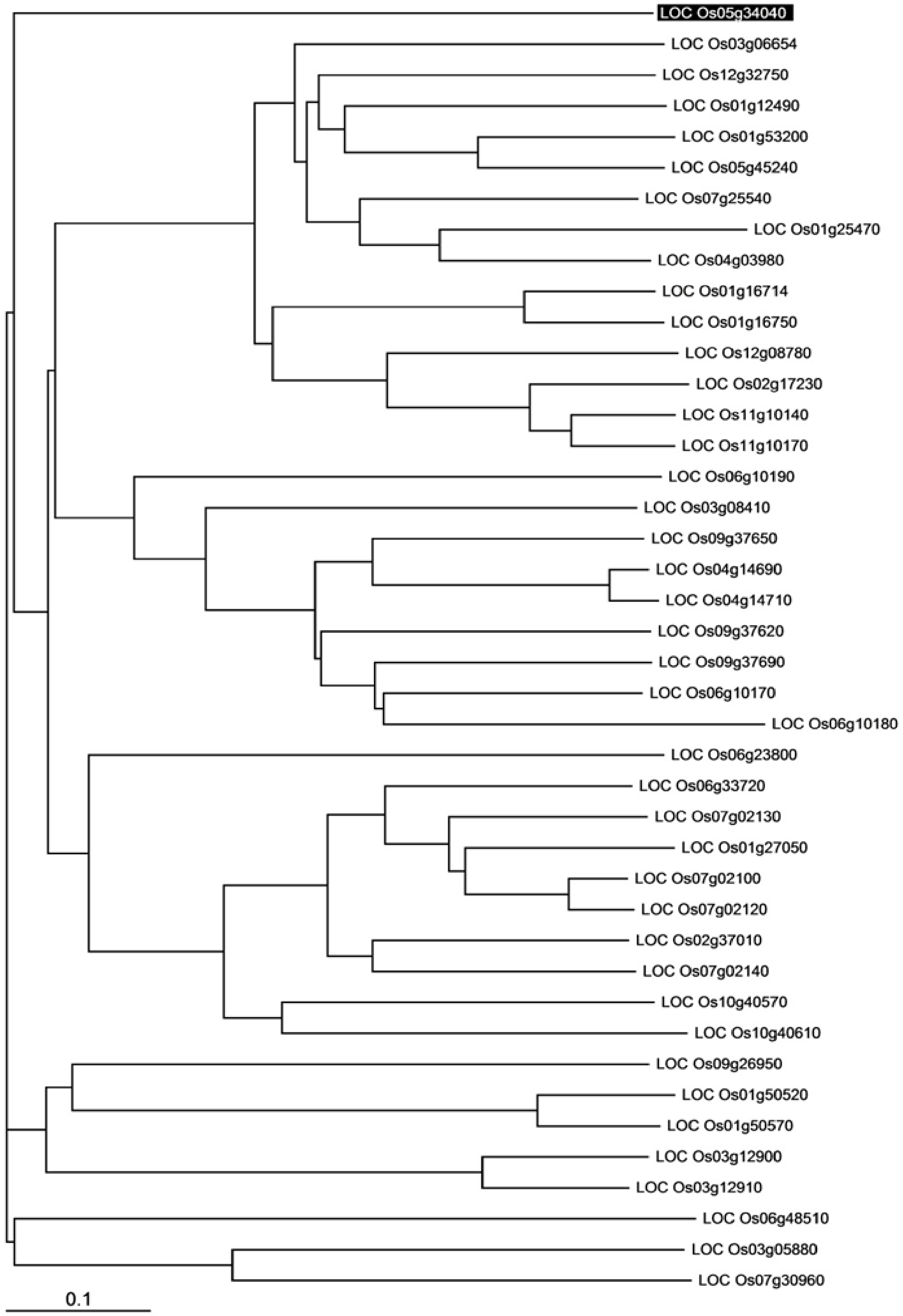

Fig. S3. Phylogenetic analysis of the TCD5 protein in rice by the neighbour-joining method.

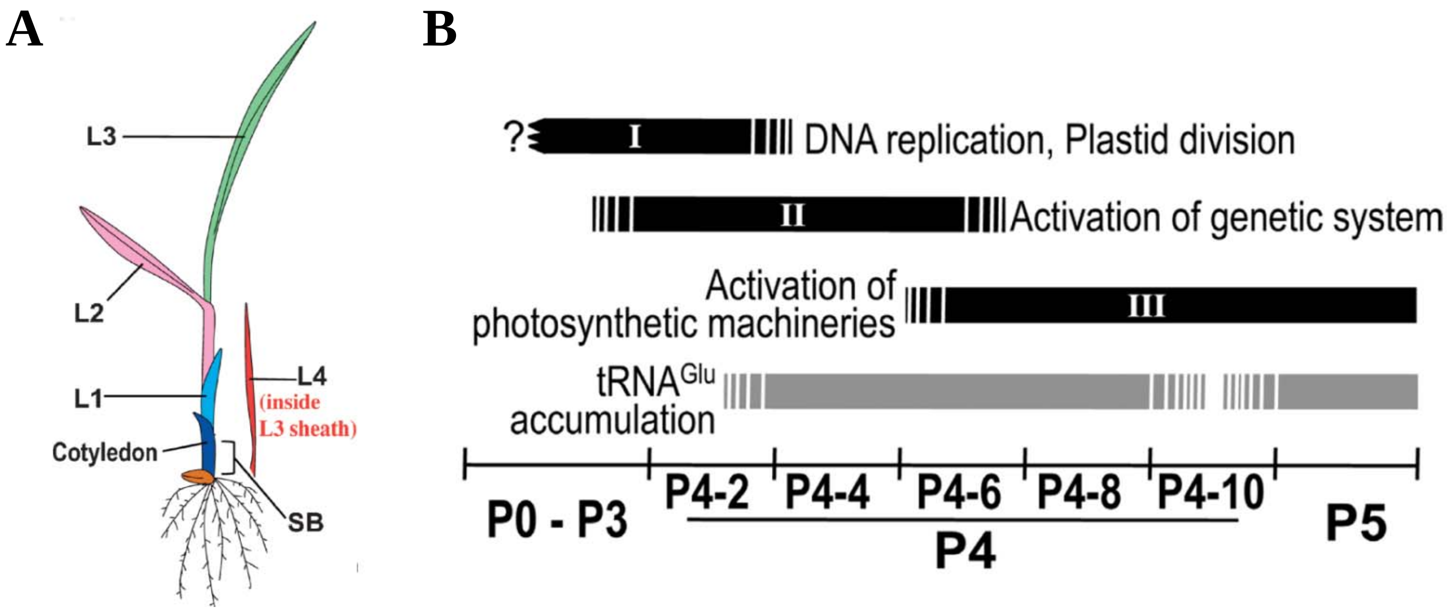

Fig. S4. Schematic illustration of a rice seedling and chronological progression of the principal growth steps during leaf development (Kusumi et al., 2010a). (A) Schematic illustration of a rice seedling with a fully expanded third leaf. L1, L2, L3 and L4 indicate the first, second, third and fourth leaves, respectively. SB, shoot base. (B) Schematic representation of the chronological progression of the principal growth steps during leaf development. Horizontal bars indicate the period when the indicated events occur in the chloroplast.
